# Maternal Metals/Metalloid Blood Levels Are Associated With Lipidomic Profiles Among Pregnant Women in Puerto Rico

**DOI:** 10.3389/fpubh.2021.754706

**Published:** 2022-01-12

**Authors:** Christine Kim, Pahriya Ashrap, Deborah J. Watkins, Bhramar Mukherjee, Zaira Y. Rosario-Pabón, Carmen M. Vélez-Vega, Akram N. Alshawabkeh, José F. Cordero, John D. Meeker

**Affiliations:** ^1^Department of Environmental Health Sciences, University of Michigan School of Public Health, Ann Arbor, MI, United States; ^2^Department of Biostatistics, University of Michigan School of Public Health, Ann Arbor, MI, United States; ^3^University of Puerto Rico Graduate School of Public Health, UPR Medical Sciences Campus, San Juan, Puerto Rico; ^4^Department of Civil and Environmental Engineering, College of Engineering, Northeastern University, Boston, MA, United States; ^5^Department of Epidemiology and Biostatistics, University of Georgia, Athens, GA, United States

**Keywords:** metals, metalloid, lipidomics, pregnancy, Puerto Rico

## Abstract

**Background/Aim:** The association between heavy metal exposure and adverse birth outcomes is well-established. However, there is a paucity of research identifying biomarker profiles that may improve the early detection of heavy metal-induced adverse birth outcomes. Because lipids are abundant in our body and associated with important signaling pathways, we assessed associations between maternal metals/metalloid blood levels with lipidomic profiles among 83 pregnant women in the Puerto Rico PROTECT birth cohort.

**Methods:** We measured 10 metals/metalloid blood levels during 24–28 weeks of pregnancy. Prenatal plasma lipidomic profiles were identified by liquid chromatography–mass spectrometry-based shotgun lipidomics. We derived sums for each lipid class and sums for each lipid sub-class (saturated, monounsaturated, polyunsaturated), which were then regressed on metals/metalloid. False discovery rate (FDR) adjusted *p*-values (*q*-values) were used to account for multiple comparisons.

**Results:** A total of 587 unique lipids from 19 lipid classes were profiled. When controlling for multiple comparisons, we observed that maternal exposure to manganese and zinc were negatively associated with plasmenyl-phosphatidylethanolamine (PLPE), particularly those containing polyunsaturated fatty acid (PUFA) chains. In contrast to manganese and zinc, arsenic and mercury were positively associated with PLPE and plasmenyl-phosphatidylcholine (PLPC).

**Conclusion:** Certain metals were significantly associated with lipids that are responsible for the biophysical properties of the cell membrane and antioxidant defense in lipid peroxidation. This study highlighted lipid-metal associations and we anticipate that this study will open up new avenues for developing diagnostic tools.

## Introduction

Lipids serve important roles in multiple processes in our body, including energy reserves and hormone regulation (1). Lipids are generally classified into eight groups, including phospholipids, glycerides, sterols, and sphingolipids (2). The lipids can be further subcategorized by their structures, such as head groups and backbone that define their specific functions. These varieties of lipids and their functions highlight the complexity of their biological roles.

The homeostasis of maternal lipid composition is critical during pregnancy, particularly for fetal development. During the first and second trimesters, there is an increase in maternal lipid levels, resulting in the accumulation of stored lipids to prepare for increased fetal energy needs in late pregnancy. However, during the third trimester, catabolism or decomposition of lipid stores occurs, as the energy demand of the fetus diminishes (3–5). Therefore, maintaining this dynamic of lipid profiles during gestation is critical not only for fetal development but also for a healthy pregnancy. For example, phosphatidylcholine (PC) is the most abundant lipid in placental tissue, as it comprises up to 36% of total lipid from healthy pregnancies (6). Previous studies have shown that any changes in the placental PC can lead to placental pathologies, such as preeclampsia (7, 8). Though the changes in the lipid profiles are considered to be physiologically normal during pregnancy, excessively elevated serum lipid levels can potentially lead to pregnancy complications, such as preterm birth and preeclampsia (9, 10).

Pregnant women are exposed to various metals and metalloids. Toxic heavy metals such as cadmium (Cd) and mercury (Hg) are known to cross the placenta (11, 12), increasing the likelihood of fetal exposure and tissue accumulation. Several previous studies have established associations between heavy metal exposures and birth outcomes, such as gestational age, birth weight, and birth height (13–17). Consistently, our previous study has shown associations between maternal metal/metalloid blood concentrations and adverse birth outcomes (18). We observed that manganese (Mn), zinc (Zn), lead (Pb), and Hg are associated with preterm birth, as well as with shorter gestational age (18). However, nickel (Ni) was associated with higher birth weight, suggesting different metals have different effects on fetal development. Several previously reported studies support our observed associations (17, 19–22). Because of the abundance of lipids in the body, particularly in the placenta, lipidomics has the potential to serve as a powerful tool to predict adverse birth outcomes by characterizing lipid profiles. Lipidomics is a study of networks of cellular lipids in the body (23, 24). Although lipids play important roles in the human biological system, particularly during pregnancy, there is a paucity of research on these potentially important biologic pathways in the relationship between metal exposures and adverse birth outcomes.

The overarching goal of this study was to investigate associations between metal exposures and lipid profiles among pregnant women in the ongoing Puerto Rico PROTECT cohort and identify lipids that may provide a better understanding of the relationship between metal exposures and adverse birth outcomes. We believe this study will contribute to identifying mechanisms by which metal exposures induce preterm birth and could inform future efforts aimed at developing diagnostic tools.

## Methods

This study sample is a subset of the Puerto Rico PROTECT cohort. The PROTECT cohort began recruitment in 2010 in the Northern Karst Region of Puerto Rico through funding from the National Institute of Environmental Health Sciences Superfund Research Program. Study participants were recruited at approximately 14 ± 2 weeks of gestation from seven prenatal clinics and hospitals throughout Northern Puerto Rico during 2010–2016. Inclusion criteria for recruitment included: participant age between 18 and 40 years; residence in the Northern Karst aquifer region; disuse of oral contraceptives 3 months before pregnancy; disuse of *in vitro* fertilization; and no indication of medical records for major obstetrical complications, including pre-existing diabetes. The blood samples were collected during the third study visit at approximately 26 weeks of gestation. From the larger cohort of 2,070 pregnant women, we randomly sampled 100 women to achieve a 1:2 case-control ratio of preterm birth, which included 31 cases and 69 controls. We created and applied inverse probability weights to account for the sampling approach (25).

### Blood Metals/Metalloid Measurements

Blood was collected in metal-free tubes, divided into aliquots, and frozen at −80°C, and shipped on dry ice to NSF International (Ann Arbor, MI, USA) for analysis. Blood concentrations of 16 metals/metalloid were measured in samples using a Thermo Fisher (Waltham, MA, USA) ICAPRQ inductively coupled plasma mass spectrometry (ICPMS) and CETAC ASX-520 autosampler, as described previously (26). Standards of known purity and identity were used during the preparation of the calibration, quality control (QC), and internal standards. The ICPMS was calibrated with a blank and a minimum of four standards for each element of interest. The calibration curve response vs. concentration was evaluated for the goodness of fit. All validated analyte correlation coefficients (*R*) were ≥0.995. Sixteen metals/metalloid were included in our analysis including arsenic (As), chromium (Cr), cadmium (Cd), nickel (Ni), copper (Cu), cobalt (Co), manganese (Mn), mercury (Hg), zinc (Zn), and iron (Fe). The resulting units were ng/ml. Specific gravity (SG) was measured using a handheld digital refractometer (Atago Co., Ltd., Tokyo, Japan) at the University of Puerto Rico Medical Sciences Campus at the time of sample collection. Metal concentrations below the limit of detection (LOD) were imputed with LOD/sqrt2.

### Lipidome Measurement

Plasma lipids were extracted from blood samples provided by women at the third visit using a modified Bligh-Dyer method using liquid–liquid extraction at room temperature after spiking with internal standards. Analysis of lipids was performed using reversed phase high-performance lipid chromatography (HPLC). This was followed by mass spectrometry (MS) analysis that alternated between MS and data dependent MS2 scans using dynamic exclusion in both positive and negative polarity, which yields excellent separation of all classes of lipids. The lipids were quantified using Multiquant and normalized by internal standards. Measurements were semi-quantitative and were based on the relative abundance of peak intensities. Quality Controls were prepared by pooling equal volumes of each sample, in addition to well-characterized plasma pools, and were injected at the beginning and end of each analysis and after every 10 sample injections, to provide a measurement of the system's stability and performance as well as reproducibility of the sample preparation methods.

Lipids were identified using the LipidBlast library (computer-generated tandem mass spectral library of 212,516 spectra covering 119,200 compounds from 26 lipid compound classes), by matching the product ions MS/MS data. The method allowed us to measure 587 lipids belonging to 19 different classes which include acylcarnitine (AcylICN), ceramides (CER), cholesterol esters (CE), diacylglycerols (DG), glucosylceramides (GlcCer), free fatty acids (FFA), fatty acid esters of hydroxyl fatty acids (FAHFA). Lysophosphatidylcholine (LysoPC), lysophophatidylethanolamine (LysoPE), phosphatidic acid (PA), phosphatidylcholine (PC), phosphatidylethanolamine (PE), phosphatidylglycerol (PG), plasmenyl-phosphatidylcholine (PLPC), plasmenyl-phosphatidylethanolamine (PLPE), phosphatidylinositol (PI), phosphatidylserine (PS), sphingomyelin (SM), and triacylglycerol (TG). All individual lipids were mentioned with the nomenclature as X:Y, where X is the length of the carbon chain and Y, the number of double bonds.

### Statistical Analysis

We explored associations between metal biomarkers and lipidomic profiles of 83 pregnant women, among which 23 were preterm, with both lipidome and metal measurements available. Inverse probability weighting of over-representation of preterm birth cases was applied to all statistical analyses presented in this analysis for our study to resemble the proportions of preterm birth in a general population (25). Ten out of 16 metals/metalloid were included in the statistical analysis as the rest of the metals/metalloid concentrations were below the LOD in >50% of samples. The group sum of the individual lipids' relative abundance that belongs to each lipid class (*n* = 19) was calculated to evaluate lipid classes. As lipid classes containing saturated, mono-unsaturated, and polyunsaturated fatty acids (PUFAs) may present different biological patterns, 45 lipid subgroups (saturated, mono-saturated, poly-unsaturated) were created by grouping lipids based on a priori knowledge of lipid class and the number of double bonds in each lipid species. Sum of lipids' relative abundance was also created for the 45 sub-groups. Descriptive statistics for lipids were computed to examine their distributions. Spearman correlation coefficients were calculated between lipid class, lipid sub-groups, and metals/metalloids to visualize the association between these groups.

Multiple statistical strategies were used to assess relationships between lipid profiles and metals/metalloids. Prior to building models, the relative abundance of individual lipids, lipid sub-group sums, and lipid group sums were log-transformed due to their skewed distribution and normalized among individuals (row) by standard normal variate (SNV) normalization. Linear regressions were then fitted to evaluate the association between metals/metalloids and the lipidome, adjusting for maternal age, maternal education, fetal sex, pre-pregnancy BMI, and weight gain during pregnancy. The final set of covariates was selected based on a priori knowledge and appreciable changes to the effect estimates from their inclusion. We investigated three hierarchical levels of lipid organization in those linear regression models: (1) individual lipidome-wide metabolites, (2) subgroup clustering based on hydrocarbon chain saturation, and (3) whole lipid classes, as explained in the following paragraph.

First, individual models were performed for each log-transformed and standardized relative abundance of lipid (*n* = 587) regressing on metals/metalloids (*n* = 10). False discovery rate (FDR) adjusted *p*-values (*q*-values), a commonly used method of adjusting for multiple comparisons in lipidomics studies, were then used to account for multiple comparisons. Sums for each lipid class (*p* = 19) and lipid sub-group (*p* = 45) (saturated, monounsaturated, polyunsaturated) were also regressed on each metals/metalloids (*n* = 10). The results are presented as estimated percentage changes of lipids, lipid group sums, sub-group sums with the corresponding 95% confidence intervals, and *p*-values per interquartile range (IQR) change in metals/metalloids.

## Results

Table 1 demonstrates the demographic and health characteristics of the study participants. The mean age of the participants at the time of enrollment was 26.5 years (standard deviation of 5.9). A majority of the participants had earned tertiary education (79.4%), household income < $50,000 (77.1%), and approximately 55% of the participants were employed at the time of enrollment. More than 50% of the study participants had a pre-pregnancy BMI of <25 kg/m^2^ (59%) and most of the participants did not smoke (97.5%) or drink (92.8%) during pregnancy.

**Table 1:** Demographic characteristics of *n* = 83 pregnant women from Puerto Rico with maternal metals/metalloid blood levels and lipidomics data available.

**Variable**	**Mean**	**SD**
Maternal age at enrollment (years)	26.5	5.9
	***N***	**%**
Maternal age (years)		
<25	36	38.7
25–30	35	37.6
>30	22	23.7
Insurance type		
Private	50	60.2
Public (Mi Salud)	29	34.9
Missing	4	4.8
Maternal education (years)		
≤ High school/GED	17	20.5
Some college or technical school	26	31.3
College degree	30	36.1
Master's degree or higher	10	12.0
Income status (US $)		
< $10,000	26	31.3
≥$10,000 to < $30,000	20	24.1
≥$30,000 to < $50,000	18	21.7
≥$50,000	8	9.6
Missing	11	13.3
Marital status		
Single	17	20.5
Married or living together	66	79.5
Gravidity (# pregnancies)		
0	42	50.6
1	28	33.7
>1	13	15.7
Pre-pregnancy BMI (kg m^−2^)		
≤ 25	49	59.0
>25– ≤ 30	16	19.3
>30	16	19.3
Missing	2	2.4
Employment status		
Employed	46	55.4
Unemployed	36	43.4
Missing	1	1.2
Smoking		
Never	71	85.5
Ever	10	12.0
Current	2	2.4
Exposure to second hand smoking		
None	69	83.1
Up to 1 h	2	2.4
More than 1 h	8	9.6
Missing	4	4.8
Alcohol consumption		
None	42	50.6
Before pregnancy	35	42.2
Yes within the last few months	5	6.0
Missing	1	1.2
Infant sex		
Female	39	47.0
Male	44	53.0

**Figure 1:**
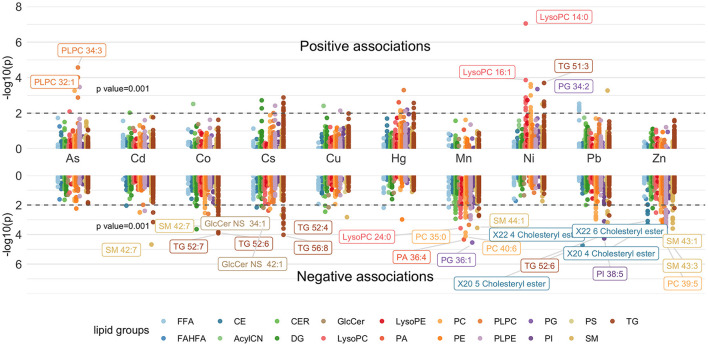
Manhattan plot showing individual lipids associated with maternal metal/metalloid blood concentrations. Models were adjusted for maternal age, maternal education, fetal sex, pre-pregnancy BMI, weight gain during pregnancy.

The associations between individual lipids and maternal metal/metalloid blood concentrations are shown by a Manhattan plot in Figure 1. Twenty-nine most significant associations after adjusting for FDR (*p*-value <0.001, *q*-value <0.05) were annotated with the individual lipid.

**Figure 2:**
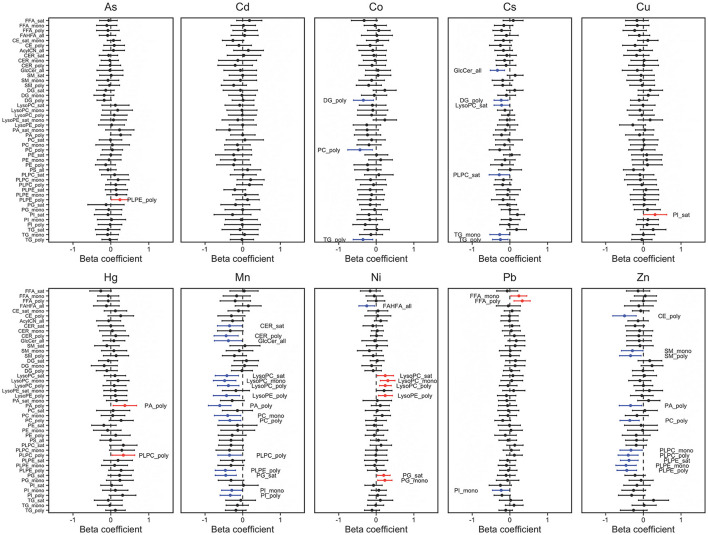
Percent change in lipid subgroup sum z-score associated with maternal metal/metalloid blood concentrations. Effect estimates presented as percent change (%) for IQR increase in exposure biomarker concentration. Models were adjusted for maternal age, maternal education, fetal sex, pre-pregnancy BMI, weight gain during pregnancy.

Based on the Manhattan plot, Ni had the greatest number of positive associations (*p* = 0.001) (Figure 1) and had the strongest association with LysoPC 14:0 (Figure 1; Supplementary Table 1). In contrast, Mn and Zn had the greatest number of negative associations and had the strongest association with PG 36:1 and SM 43:3, respectively (Figure 1; Supplementary Table 1). The associations between lipid subgroups based on the degree of saturation and maternal metal/metalloid blood concentrations are further shown in a forest plot in Figure 2. Significant associations after FDR correction (*p*-value < 0.001 and *q*-value < 0.05) were annotated with the lipid subgroups with red representing a positive association and blue indicating a negative association. Ni, Cu, As, and Hg had significant positive associations with the lipid subgroups. Consistent with the Manhattan plot, Ni had the strongest positive association, specifically with mono-unsaturated LysoPC (Figure 2; Supplementary Table 2). In contrast, Co, Cs, Mn, Pb, and Zn had significant negative associations with the lipid subgroups. Among these associations, Mn and Zn had the greatest number of negative associations. The lipid subgroups that were mainly associated with Mn and Zn were mono-unsaturated and poly-unsaturated LysoPC and poly-unsaturated, mono-unsaturated, and saturated PLPE, respectively (Figure 2; Supplementary Table 2).

**Figure 3:**
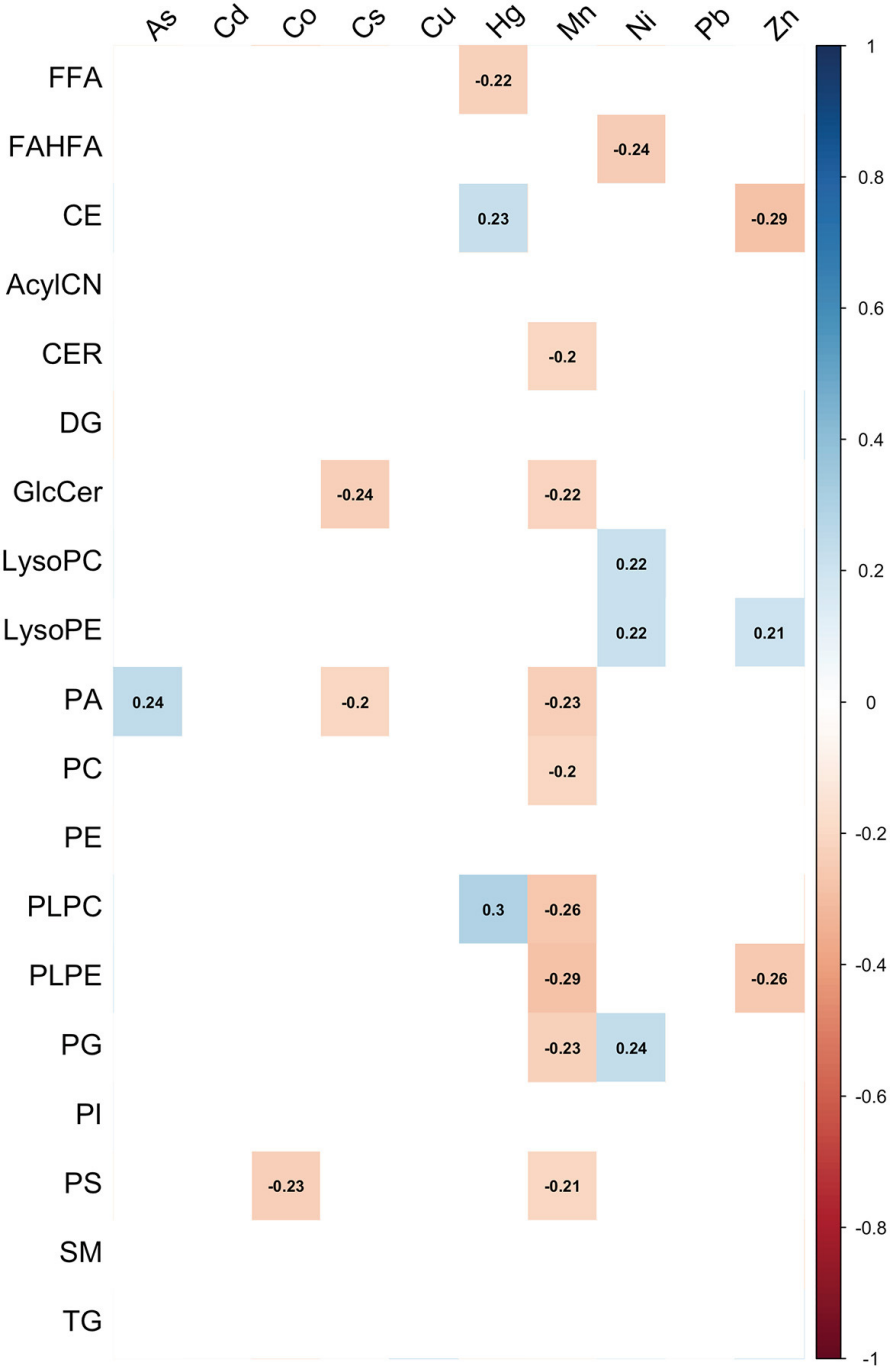
Correlation matrix between maternal metal/metalloid blood concentrations and lipid classes.

**Figure 4:**
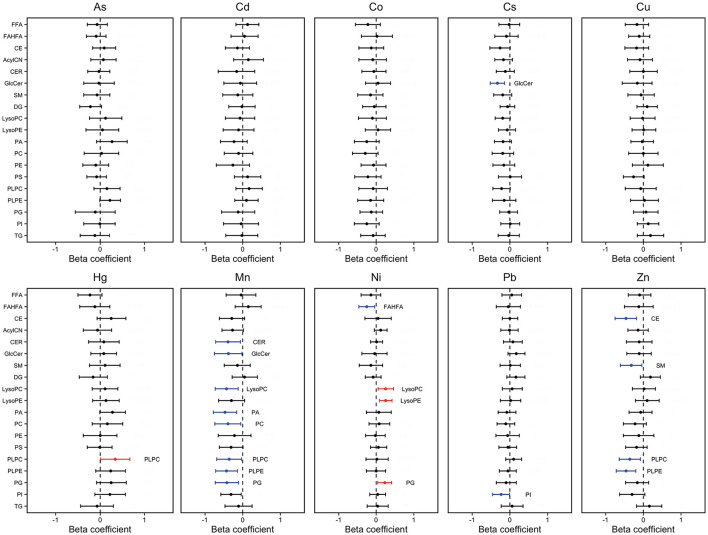
Percent change in lipid class sum z-score associated with maternal metal/metalloid blood concentrations. Effect estimates presented as percent change (%) for IQR increase in exposure biomarker concentration^ab^. Models were adjusted for maternal age, maternal education, fetal sex, pre-pregnancy BMI, weight gain during pregnancy.

The heat map in Figure 3 shows the relationship between maternal metal/metalloid blood concentrations and lipid groups. Spearman correlation coefficients ranged from −0.29 to 0.3. Consistent with Figures 1, 2, both Mn and Zn had significant negative associations with the lipid groups (Supplementary Table 3). Mn had the strongest negative associations with PLPC (0.26) and PLPE (−0.29) (Figure 3). Conversely, Zn had the most negative associations with PLPE (−0.26) and CE (−0.29). Furthermore, As, Hg, and Ni had the strongest positive associations with PA (0.24), PLPC (0.3), and PG (0.24), respectively (Figure 3). These observed associations are further supported by a forest plot demonstrated in Figure 4.

## Discussion

Adverse birth outcomes related to metal exposures are a global issue. In our previous study, we observed significant associations between maternal Mn and Zn blood levels with preterm birth in the PROTECT cohort (18). Because the homeostasis of maternal lipid composition is critical during pregnancy, our current study focused on identifying lipid markers to explore lipid–metal associations to obtain a better understanding of the relationship between metal exposures and adverse birth outcomes and contribute to the development of diagnostic tools for predicting adverse birth outcomes.

First, we assessed the association between individual lipid metabolites and maternal metal/metalloid blood levels. Ni had the greatest number of positive associations and had the strongest association with LysoPC 14:0 (Figure 1; Supplementary Table 1). Consistently, Ni had a significant positive association with mono-unsaturated LysoPC (Figure 2). It is important to note that LysoPC has an anti-migratory ability of endothelial cells (27), which can be found in all blood vessels. Angiogenesis is the formation of blood vessels, and it involves the migration of endothelial cells (28), and importantly, it is a critical process during normal pregnancy (29). Without the invasion of endothelial cells, the primitive vascular network that is required for proper fetal development is impaired. In a recent study, Chen et al. found a significant association between maternal exposure to Ni and preterm birth, as well as decreased gestational age (30). Consistently, Horgan et al. observed an association between total maternal plasma LysoPC level and small for gestational age (SGA) at 15 weeks gestation (31). Together, these studies support our observations and suggest a possible interplay between lipids and metals and its relation to adverse birth outcomes.

Conversely, Zn and Mn had the greatest number of negative associations with the individual lipid metabolites and had significant associations with SM 43:3 and PG 36:1, respectively (Figure 1; Supplementary Table 1). It is important to highlight that we have observed associations of maternal blood Mn and Zn levels with preterm birth, as well as SGA (18). Though our previous study has demonstrated a significant association between PG 36:1 and increased gestational age (32), there is no clear evidence that suggests a link between SM 43:3 and adverse birth outcomes. A recent study, however, has demonstrated the role of SM on regulating placental vascularization and facilitating placental-uterine invasion (33), suggesting the impact of lipids on birth outcomes. Next, we observed significant negative associations of Mn with poly-unsaturated PA and poly-unsaturated PLPE, and Zn with poly-unsaturated PLPE (Figure 2; Supplementary Table 2). Though a paucity of research on plasmenyl phospholipids highlights the complexity in the interpretation of our results, in our previous study, we observed low to moderate positive associations of poly-unsaturated PA and poly-unsaturated PLPE with large for gestational age (LGA), as well as low to moderate negative associations with SGA (32). Taken together, these observations further provide evidence of a possible interplay between metals and lipids and its relation to adverse birth outcomes.

Lastly, we observed the associations between maternal blood metal/metalloid levels and whole lipid class. Consistently, we observed significant negative associations of PLPE with maternal Mn and Zn blood levels (Figures 3, 4). Despite the strong inverse correlations, there is no clear mechanism to explain the observed inverse association between maternal Mn and Zn blood levels with PLPE. Conversely, maternal As and Hg blood levels had positive associations with PLPC and PLPE (Figures 3, 4). It is important to note that metals can trigger different signaling pathways and regulators (34). Our previous study suggests that an increased circulating plasmenyl phospholipid, specifically PLPE, is linked to spontaneous preterm birth and LGA (32). Mn, Zn, As, and Hg are all linked to preterm birth (18, 35–38), but, based on our observations, they have the opposite associations with plasmenyl phospholipid that is strongly linked to spontaneous preterm birth and LGA. Some studies have reported conflicting results by demonstrating increased cleavage of plasmenyl phospholipids from Hg exposure (39, 40). These conflicting results arise in part because of several inherent limitations, such as within- and between-cohort variation, sensitivity to different species of metals, and different exposure levels. Unfortunately, a paucity of research on plasmenyl phospholipid–metal interactions limits our understanding of how metals differentially impact plasmenyl phospholipids. Additional studies, such as assessing maternal blood metal/metalloid levels and plasmenyl phospholipids in all three stages of pregnancy will provide more insight into the role of metal exposures in adverse birth outcomes.

There are important limitations in our study. One limitation is the modest sample size. However, this is an exploratory study and future investigations with a larger sample size could provide a better understanding of lipid–metal associations. Furthermore, the majority of the previously published lipidomic studies had used smaller sample sizes. Another limitation in our study design is that we did not use placental tissue to measure the lipids. By using the placental tissue, we would be able to measure the lipids directly from the placenta to better assess the impact of heavy metals on placental lipid groups. Also, it is important to note that the blood sample that was used in this study was obtained at the third visit (~26 weeks gestation). Lipid profile changes dramatically during pregnancy. Therefore, using a blood sample only from a single time point prevents us from looking at the dynamic changes of lipids over time. Because decomposition of lipid storage occurs during the third trimester, there is a possibility that lipid profiles will be different from earlier time points. Lastly, our study focused on an underrepresented community in the U.S., therefore, the generalizability of these observations to other populations may be limited.

Despite these limitations, it is important to highlight the strengths of our study. One of the strengths of this study is that these lipids and metal/metalloid levels can be measured in blood, a commonly collected matrix. Another strength of our study is that there is limited research to date on the associations between metal exposure and lipidomics in the context of pregnancy and adverse birth outcomes. Finally, we conducted this preliminary analysis in an established and well-characterized birth cohort among an underrepresented population of pregnant women at risk for elevated environmental exposures as well as adverse birth outcomes.

In this exploratory study, we present new information that may contribute to the early detection of adverse birth outcomes in pregnant women who are exposed to environmental contaminants. We observed both positive and negative associations between maternal metal/metalloid blood levels and lipid profiles in pregnant mothers in the PROTECT cohort. Most importantly, we observed strong negative associations of Mn and Zn with PLPE. Taken together, these findings highlight the need to further assess not only lipid-metal associations but also plasmenyl phospholipid–metal interactions and their relation to adverse birth outcomes. Also, future work is warranted to explore the association between fetal sex-specific lipid profiles and metal exposures to substantiate the present study.

## Data Availability Statement

The original contributions presented in the study are included in the article/Supplementary Material, further inquiries can be directed to the corresponding author/s.

## Ethics Statement

The studies involving human participants were reviewed and approved by University of Michigan School of Public Health, University of Puerto Rico, and Northeastern University. The patients/participants provided their written informed consent to participate in this study.

## Author Contributions

CK: writing—review and editing. PA: formal analysis, investigation, methodology, and editing. DW: conceptualization, editing, and funding acquisition. BM: conceptualization, supervision, and funding acquisition. ZR-P and CV-V: data curation and project administration. AA and JC: conceptualization and funding acquisition. JM: conceptualization, funding acquisition, and supervision. All authors contributed to the article and approved the submitted version.

## Funding

This study was supported by the Superfund Research Program of the National Institute of Environmental Health Sciences, National Institutes of Health (P42ES017198). Additional support was provided from NIEHS grant numbers T32ES007062, P50ES026049, R01ES032203, and P30ES017885 and the Environmental influences on Child Health Outcomes (ECHO) program grant number UH3OD023251.

## Conflict of Interest

The authors declare that the research was conducted in the absence of any commercial or financial relationships that could be construed as a potential conflict of interest.

## Publisher's Note

All claims expressed in this article are solely those of the authors and do not necessarily represent those of their affiliated organizations, or those of the publisher, the editors and the reviewers. Any product that may be evaluated in this article, or claim that may be made by its manufacturer, is not guaranteed or endorsed by the publisher.
