# Supplementary material for: Maternal Metals/Metalloid Blood Levels Are Associated With Lipidomic Profiles Among Pregnant Women in Puerto Rico
Source: Front Public Health. 2022 Jan 12;9:754706. doi: 10.3389/fpubh.2021.754706 (PMC8790322; doi:10.3389/fpubh.2021.754706)
Supplement: Supplementary file 1 [file Table_1.DOCX]

**Maternal Metals/Metalloid Blood Levels are Associated with Lipidomic Profiles Among Pregnant Women in Puerto Rico.**

Christine Kim^1^, Pahriya Ashrap^1^, Deborah J. Watkins^1^, Bhramar Mukherjee^2^, Zaira Y. Rosario-Pabón^3^, Carmen M. Vélez-Vega^3^, Akram N. Alshawabkeh^4^, José F. Cordero^5^, John D. Meeker^1^

^1^University of Michigan School of Public Health, Department of Environmental Health Sciences, Ann Arbor, Michigan, United States

^2^University of Michigan School of Public Health, Department of Biostatistics, Ann Arbor, Michigan, United States

^3^University of Puerto Rico Graduate School of Public Health, UPR Medical Sciences Campus, San Juan, Puerto Rico

^4^College of Engineering, Northeastern University, Boston, Massachusetts, United States

^5^Department of Epidemiology and Biostatistics, University of Georgia, Athens, Georgia, United States

*Corresponding author: John D. Meeker, University of Michigan School of Public Health, Department of Environmental Health Sciences, 1415 Washington Heights, Ann Arbor, Michigan 48109, USA, email: [meekerj@umich.edu](mailto:meekerj@umich.edu), telephone: 1.734.764.7184

**Supplementary Table 1**. The top individual lipids associated with maternal metal/metalloid blood concentrations.

| **Lipids** | **Lipid Class Name** | **Metal** | **p1** | **q1** |
| --- | --- | --- | --- | --- |
| LysoPC.14.0 | LysoPC | Ni | 8.97E-08 | 2.06E-05 |
| X20.5.Cholesteryl.ester | CE | Pb | 1.89E-05 | 2.64E-03 |
| SM.43.3 | SM | Zn | 1.98E-05 | 7.18E-03 |
| SM.42.7 | SM | Cd | 2.11E-05 | 7.18E-03 |
| Plasmenyl.PC.34.3 | PLPC | As | 2.69E-05 | 1.48E-02 |
| PG.36.1 | PG | Mn | 2.86E-05 | 2.00E-03 |
| PA.36.4 | PA | Mn | 4.62E-05 | 2.77E-03 |
| PI.38.5 | PI | Pb | 5.44E-05 | 7.07E-03 |
| PC.40.6 | PC | Mn | 7.76E-05 | 3.94E-02 |
| TG.56.8 | TG | Cs | 9.54E-05 | 3.88E-02 |
| Plasmenyl.PC.32.1 | PLPC | As | 9.78E-05 | 2.69E-02 |
| PC.39.5 | PC | Zn | 1.07E-04 | 3.94E-02 |
| TG.52.7 | TG | Co | 1.21E-04 | 3.88E-02 |
| TG.52.6 | TG | Pb | 1.35E-04 | 3.88E-02 |
| PC.35.0 | PC | Mn | 1.36E-04 | 3.94E-02 |
| LysoPC.16.1 | LysoPC | Ni | 1.36E-04 | 1.57E-02 |
| TG.52.6 | TG | Co | 1.49E-04 | 3.88E-02 |
| TG.51.3 | TG | Ni | 2.00E-04 | 4.15E-02 |
| TG.52.4 | TG | Cs | 2.54E-04 | 4.40E-02 |
| SM.43.1 | SM | Zn | 2.55E-04 | 4.75E-02 |
| LysoPC.24.0 | LysoPC | Mn | 2.68E-04 | 2.05E-02 |
| SM.44.1 | SM | Mn | 2.82E-04 | 4.75E-02 |
| SM.42.7 | SM | Co | 3.50E-04 | 4.75E-02 |
| X20.4.Cholesteryl.ester | CE | Zn | 3.73E-04 | 2.29E-02 |
| GlcCer.NS..42.1 | GlcCer | Cs | 4.27E-04 | 2.73E-02 |
| PG.34.2 | PG | Ni | 4.41E-04 | 1.54E-02 |
| X22.6.Cholesteryl.ester | CE | Zn | 4.91E-04 | 2.29E-02 |
| GlcCer.NS..34.1 | GlcCer | Cs | 5.46E-04 | 2.73E-02 |
| X22.4.Cholesteryl.ester | CE | Zn | 8.24E-04 | 2.89E-02 |

Abbreviations: lysophosphatidylcholine (LysoPC); sphingomyelin (SM); phosphatidylcholine (PC); phosphatidylglycerol (PG); phosphatidic acid (PA); phosphatidylinositol (PI); triacylglycerol (TG); glucosylceramides (GlcCer); cholesterol esters (CE); plasmenyl-phosphatidylcholine (PLPC); cobalt (Co); cesium (Cs); copper (Cu); manganese (Mn); nickel (Ni); zinc (Zn); arsenic (As); cadmium (Cd); lead (Pb).

**Supplementary Table 2**. The top lipid subgroups associated with maternal metal/metalloid blood concentrations.

| **Lipid Subgroup** | **Lipid Class Name** | **Metal** | **p1** | **q1** |
| --- | --- | --- | --- | --- |
| PA_poly | PA | Mn | 1.57E-04 | 1.57E-03 |
| PLPE_poly | PLPE | Mn | 1.43E-03 | 7.56E-03 |
| PLPE_poly | PLPE | Zn | 1.51E-03 | 7.56E-03 |
| GlcCer_all | GlcCer | Cs | 1.16E-03 | 1.16E-02 |
| PLPE_mono | PLPE | Zn | 1.27E-03 | 1.27E-02 |
| LysoPC_mono | LysoPC | Mn | 2.56E-03 | 1.76E-02 |
| LysoPC_mono | LysoPC | Ni | 3.51E-03 | 1.76E-02 |
| CE_poly | CE | Zn | 2.20E-03 | 2.20E-02 |
| PG_sat | PG | Mn | 2.22E-03 | 2.22E-02 |
| PLPE_sat | PLPE | Zn | 2.24E-03 | 2.24E-02 |
| LysoPC_poly | LysoPC | Ni | 2.85E-03 | 2.85E-02 |
| FFA_poly | FFA | Pb | 3.46E-03 | 3.46E-02 |
| PLPC_poly | PLPC | Zn | 3.91E-03 | 3.91E-02 |
| LysoPC_poly | LysoPC | Mn | 9.73E-03 | 4.87E-02 |

Abbreviations: lysophosphatidylcholine (LysoPC); phosphatidylglycerol (PG); phosphatidic acid (PA); glucosylceramides (GlcCer); cholesterol esters (CE); plasmenyl-phosphatidylcholine (PLPC); plasmenyl-phosphatidylethanolamine (PLPE); free fatty acids (FFA); cesium (Cs); manganese (Mn); nickel (Ni); zinc (Zn).

**Supplementary Table 3**. The top lipid groups associated with maternal metal/metalloid blood concentrations.

| **Lipid Class Name** | **Lipid Class Full Name** | **Metal** | **p1** | **q1** |
| --- | --- | --- | --- | --- |
| PLPE | Plasmenyl phosphatidylethanolamine | Zn | 9.54E-04 | 9.54E-03 |
| GlcCer | Glucosylceramides | Cs | 1.16E-03 | 1.16E-02 |
| CE | Cholesterol esters | Zn | 2.15E-03 | 2.15E-02 |
| PLPE | Plasmenyl phosphatidylethanolamine | Mn | 5.22E-03 | 2.61E-02 |
| LysoPE | Lysophosphatidylethanolamine | Ni | 3.03E-03 | 3.03E-02 |
| PA | Phosphatidic acid | Mn | 4.40E-03 | 4.40E-02 |

Abbreviations: lysophosphatidylethanolamine (LysoPE); phosphatidic acid (PA); glucosylceramides (GlcCer); cholesterol esters (CE); plasmenyl-phosphatidylethanolamine (PLPE); cesium (Cs); manganese (Mn); nickel (Ni); zinc (Zn).
